# 2519. A Multi-national Phase 3, Randomized, Double-Blind, Active Comparator-Controlled Clinical Trial to Study the Safety, Tolerability, and Efficacy of Imipenem/Cilastatin/Relebactam (MK-7655A) Versus Piperacillin/Tazobactam in Subjects with Hospital-Acquired Bacterial Pneumonia or Ventilator-Associated Bacterial Pneumonia

**DOI:** 10.1093/ofid/ofad500.2137

**Published:** 2023-11-27

**Authors:** Junjie Li, Feng Wei, Peng Xiang, Zhengang Tang, Lianshu Ding, Maria C Losada, Zlatka Iamboliyska, Mingfen Zhu, Xiaodan Guo, Xiaoling Du, Chang Chen, Luke Francis Chen, Min Zhou, Jieming Qu

**Affiliations:** Ruijin Hospital, Shanghai Jiaotong University School of Medicine, Shanghai, China; Institute of Respiratory Diseases, Shanghai Jiaotong University School of Medicine, Shanghai, China;, N/A, Shanghai, China; The First People’s Hospital of Nanning, Nanning, China, Nanning, Guangxi, China; Changsha Central Hospital, China, Changsha, Hunan, China; Shiyan Renmin Hospital, Shiyan, China, Shiyan, Hubei, China; The Affiliated Huai'an First Hospital of Nanjing Medical University, Huai'an, China, Huaian, Jiangsu, China; Merck & Co., Inc., Rahway, New Jersey; Merck & Co., Inc., Rahway, New Jersey, USA, South San Francisco CA, California; MSD, China, Xuhui district, Shanghai, China; MSD, China, Xuhui district, Shanghai, China; MSD, China, Xuhui district, Shanghai, China; MSD, China, Xuhui district, Shanghai, China; Merck & Co., Inc., Rahway, New Jersey, USA, South San Francisco CA, California; Ruijin Hospital, Shanghai Jiaotong University School of Medicine, Shanghai, China; Institute of Respiratory Diseases, Shanghai Jiaotong University School of Medicine, Shanghai, China, N/A, Shanghai, China; Ruijin Hospital, Shanghai Jiaotong University School of Medicine, Shanghai, China; Institute of Respiratory Diseases, Shanghai Jiaotong University School of Medicine, Shanghai, China, N/A, Shanghai, China

## Abstract

**Background:**

Hospital-acquired bacterial pneumonia (HABP) and ventilator-associated bacterial pneumonia (VABP) are associated with high mortality rates. In these patients, there is an unmet need for effective antimicrobial therapy. This study enrolled participants mainly from China to support the regional use of imipenem/cilastatin/relebactam (IMI/REL). The aim of the study was to assess the efficacy and safety of IMI/REL compared with piperacillin/tazobactam (PIP/TAZ) for the treatment of HABP/VABP.

**Methods:**

This study was a Phase 3, randomized trial conducted in 8 countries (NCT03583333). Participants 18–90 years of age with HABP/VABP requiring intravenous (IV) antibiotic treatment were randomized 1:1 to receive either IV IMI/REL (500 mg IMI/250 mg REL) or PIP/TAZ (4000 mg/500 mg) administered every 6 h for 7–14 days. The primary endpoint was day 28 all-cause mortality in the modified intent-to-treat (MITT) population (excluding patients with only gram-positive cocci at baseline). Secondary endpoints included clinical response rate, microbiological response rate and adverse events (AEs).

**Figure:**
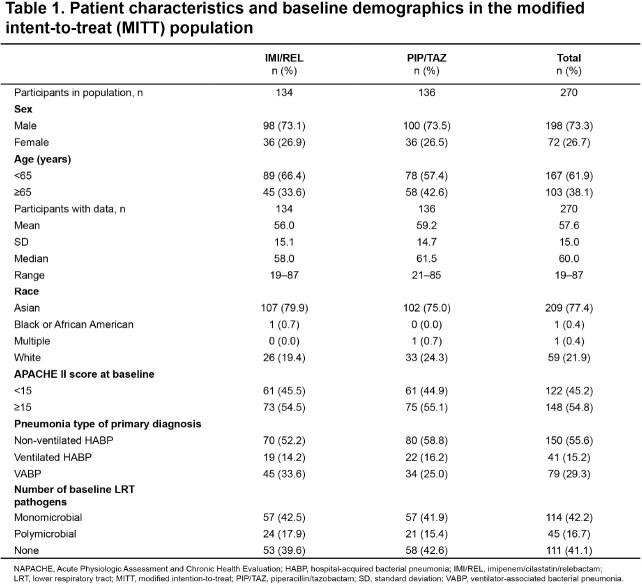

**Results:**

In total, 270 patients (IMI/REL, N=134; PIP/TAZ, N=136) were included in the MITT population, with 201 patients from mainland China (IMI/REL, N=106; PIP/TAZ, N=100). Demographics and baseline characteristics were generally comparable for both treatment groups in the MITT population, with the exception of preexisting cerebrovascular conditions which were higher in IMI/REL vs PIP/TAZ group. The median age (range) was 60.0 (19–87) years and 73.3% were male (Table 1). In the MITT population, IMI/REL was non-inferior to PIP/TAZ for day 28 all-cause mortality (11.2% vs 5.9%; adjusted difference [95%CI]: 5.2% [-1.5%, 12.4%],P< 0.024). Results for key secondary endpoints were comparable between treatment groups (Table 2). Overall, the incidence of AEs was comparable between IMI/REL vs PIP/TAZ (Table 3).

**Figure:**
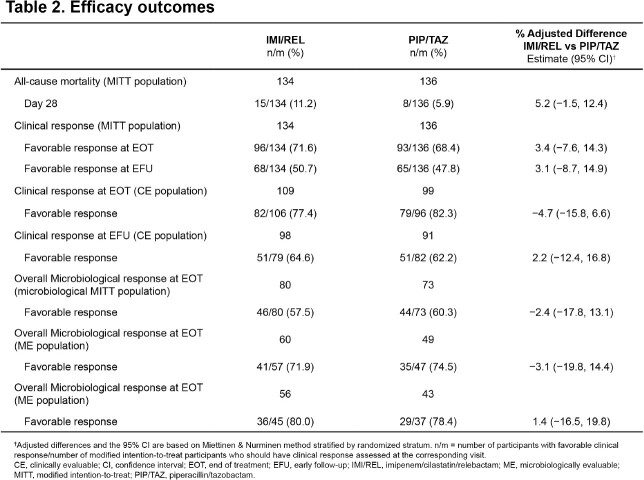

**Figure:**
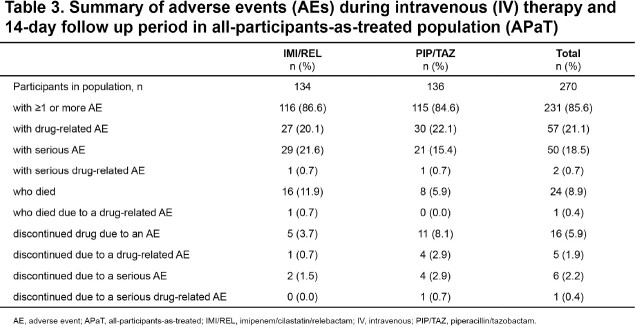

**Conclusion:**

In a patient population mainly from China, IMI/REL was non-inferior to PIP/TAZ for the treatment of HABP/VABP assessed by the endpoint of day 28 all-cause mortality and was associated with favorable outcomes regarding clinical and microbiological response.IMI/REL was well tolerated and had a safety profile comparable to PIP/TAZ.

**Disclosures:**

**Maria C. Losada, BA**, Merck & Co., Inc., Rahway, New Jersey, USA: Employee|Merck & Co., Inc., Rahway, New Jersey, USA: Stocks/Bonds|Merck & Co., Inc., Rahway, New Jersey, USA: Stocks/Bonds **Zlatka Iamboliyska, MD**, MSD China: Employee|MSD China: Stocks/Bonds **Mingfen Zhu, MD**, MSD China: Employee|MSD China: Stocks/Bonds **Xiaodan Guo, MD**, MSD China: Employee|MSD China: Stocks/Bonds **Xiaoling Du, n/a**, MSD China: Employee|MSD China: Stocks/Bonds **Chang Chen, MD**, MSD China: Employee|MSD China: Stocks/Bonds **Luke Francis Chen, MD**, Merck & Co., Inc., Rahway, New Jersey, USA: Employee|Merck & Co., Inc., Rahway, New Jersey, USA: Stocks/Bonds

